# Epidemiology of Hospitalizations Associated with Invasive Candidiasis, United States, 2002–2012^1^

**DOI:** 10.3201/eid2301.161198

**Published:** 2017-01

**Authors:** Sara Strollo, Michail S. Lionakis, Jennifer Adjemian, Claudia A. Steiner, D. Rebecca Prevots

**Affiliations:** National Institutes of Health, Bethesda, Maryland, USA (S. Strollo, M.S. Lionakis, J. Adjemian, D.R. Prevots);; United States Public Health Service, Rockville, Maryland, USA (J. Adjemian);; Agency for Healthcare Research and Quality, Rockville (C.A. Steiner)

**Keywords:** epidemiology, invasive candidiasis, candidemia, Candida spp., fungi, fungal infections, opportunistic infections, hospitalizations, incidence, United States

## Abstract

Invasive candidiasis is a major nosocomial fungal disease in the United States associated with high rates of illness and death. We analyzed inpatient hospitalization records from the Healthcare Cost and Utilization Project to estimate incidence of invasive candidiasis–associated hospitalizations in the United States. We extracted data for 33 states for 2002–2012 by using codes from the International Classification of Diseases, 9th Revision, Clinical Modification, for invasive candidiasis; we excluded neonatal cases. The overall age-adjusted average annual rate was 5.3 hospitalizations/100,000 population. Highest risk was for adults >65 years of age, particularly men. Median length of hospitalization was 21 days; 22% of patients died during hospitalization. Median unadjusted associated cost for inpatient care was $46,684. Age-adjusted annual rates decreased during 2005–2012 for men (annual change –3.9%) and women (annual change –4.5%) and across nearly all age groups. We report a high mortality rate and decreasing incidence of hospitalizations for this disease.

## Abstract 2

Highest hospitalization rates were for men ≥65 years of age, and rates decreased during 2005−2012.

Opportunistic fungi are a major cause of invasive nosocomial infections, particularly among patients with long-term stays in intensive care units; central venous catheters; recent surgery; and immunosuppression, such as those with hematopoietic stem cell transplantation and hematologic malignancies (*1**–**4*). *Candida* species are associated with invasive fungal infections among at-risk groups and have been ranked seventh as a cause of nosocomial bloodstream infection in the United States and elsewhere (*4**–**6*). These fungi are common gastrointestinal flora that cause a wide range of severe manifestations when disseminated into the bloodstream. Although candidemia has been described as the most common manifestation of invasive candidiasis, deep-seated infections of organs or other sites, such as the liver, spleen, heart valves, or eye, might also occur after a bloodstream infection and persist after clearance of fungi from the bloodstream (*1**,**7*).

Candidemia is associated with high rates of illness and death and has an attributable mortality rate >30%–40% in the United States (*8*). However, unadjusted mortality rates vary widely in the literature, ranging from 29% to 76% (*3**,**8**–**13*). Increased hospital costs and prolonged length of stay associated with invasive candidiasis contribute to a major financial burden, which is believed to exceed 2 billion dollars in the United States per year (*14*).

A population-based study of candidemia in the United States with active laboratory surveillance data for 2 cities (Atlanta, Georgia, and Baltimore, Maryland) reported incidences in these areas and a major decrease during 2008–2013 (*13**,**15*). However, current nationally representative data with state-specific estimates for the United States are lacking. To provide a more complete and current picture of the epidemiology of invasive candidiasis, including state-specific prevalence of hospitalizations, geographic patterns, and cost, we analyzed nationally representative hospital discharge data for this disease.

## Methods

### Data Source and Study Population

We extracted data from the State Inpatient Databases maintained by the US Agency for Healthcare Research and Quality (AHRQ) through the Healthcare Cost and Utilization Project (*16*). This project was conducted through an active collaboration between the National Institutes of Health (Bethesda, MD, USA) and the AHRQ Healthcare Cost and Utilization Project. As of 2014, the SID included 48 participating states and encompassed 97% of all US community hospital discharges.

Inpatient hospital discharge records were extracted by using codes from the International Classification of Diseases, 9th revision, Clinical Modification (ICD-9-CM), for invasive candidiasis, specifically those records with disseminated candidiasis (code 112.5), candidal endocarditis (code 112.81), and candidal meningitis (code 112.83) listed anywhere in primary or secondary diagnostic fields. All secondary diagnostic fields are those other than primary fields and have 1–29 additional diagnostic codes. We excluded records with ICD-9-CM codes for localized *Candida* species infections. In addition, to avoid misclassification of noninvasive neonatal candidiasis as invasive candidiasis, we excluded records with codes for neonatal candidiasis (code 771.7), and records for infants <1 month (28 days) of age.

Our analysis covered 33 states that had complete demographic data and continuous participation during 2002–2012; these states contain ≈81% of the US population. Variables collected for each discharge record included year of admission, state of hospitalization, age at admission, sex, length of hospitalization, ICD-9 code (primary discharge diagnosis and up to 29 secondary codes), in-hospital deaths, and hospitalization cost.

### Data Analysis

We used US Census Bureau age-, sex-, and race-specific state population data as denominators for all hospitalization rate calculations. For national and state estimates, age-adjusted hospitalization rates were calculated by using the US Census 2010 population as the reference population. Primary and secondary discharge codes among all records with an invasive candidiasis–associated hospitalization were analyzed to identify relevant concurrent conditions or procedures. Of the abstracted hospitalization records, 93% had 9 diagnostic codes. AHRQ Clinical Classification Software was used to collapse ICD-9-CM codes into a smaller number of clinically meaningful categories for analyzing concurrent conditions (*17*).

To estimate economic burden, we used total hospital costs for 15 states with publically available cost-to-charge data during 2002–2012, which represent 36% of the US population. Total cost of hospital stay was converted from hospitalization charge by using AHRQ cost-to-charge ratio files specific for hospital groups (*18*). Total hospital costs approximate the cost of providing the inpatient service, excluding physician services, and have been shown to better represent economic effect than inpatient charges (*19*). Medical Care Consumer Price Index data from the Bureau of Labor Statistics were used to adjust nominal estimated costs to reflect constant 2015 US dollars (*20*). All costs are presented in US dollars.

We analyzed a subset of 23 states with continuous race reporting during 2002–2012, which were representative of 61% of the US population, to describe hospitalizations by race. For trend analysis, we estimated the average annual percent change (APC) from Poisson regression models and used prevalence as the dependent variable and time (year) as the independent variable. Separate models were also fit for each age stratum. A p value <0.05 was considered statistically significant. SEs were scaled by using the Pearson χ^2^ statistic to account for overdispersion. All analysis was conducted by using SAS version 9.3 (SAS Institute, Cary, NC, USA).

## Results

**Figure 1:**
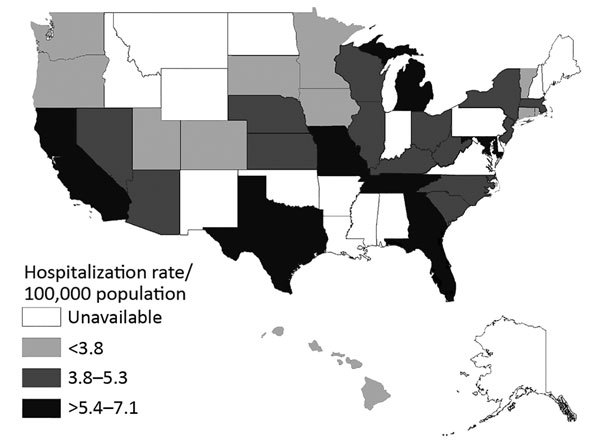
Average annual invasive candidiasis−associated hospitalizations, United States, 2002−2012. Data were provided by State Inpatient Databases through the Healthcare Cost and Utilization Project maintained by the US Agency for Healthcare Research and Quality. Diagnoses were classified by using Agency for Healthcare Research and Quality clinical classification software (*17*) and multiple codes and ranges from the International Classification of Diseases, 9th Revision, Clinical Modification.

During 2002–2012, we identified 138,433 invasive candidiasis–associated hospital discharges (average annual age-adjusted hospitalization rate 5.3 hospitalizations/100,000 population). Overall, 97% (134,225/138,433) of invasive candidiasis–associated hospitalizations were coded as disseminated candidiasis, 3% (4,253) as candidal endocarditis, and 1% (1,321) as candidal meningitis. Over the 11-year period, 1% (1,366 discharges) of hospitalization records were coded for disseminated candidiasis and candidal endocarditis or candidal meningitis; 16% (22,151 discharges) had an invasive candidiasis code as the primary diagnosis. State-specific, age-adjusted, average annual hospitalizations per 100,000 population ranged from a low of 2.0 in Vermont to 7.1 in Maryland (Figure 1). Temporal trends were similar across states, and no clear regional patterns among states were observed.

**Figure 2:**
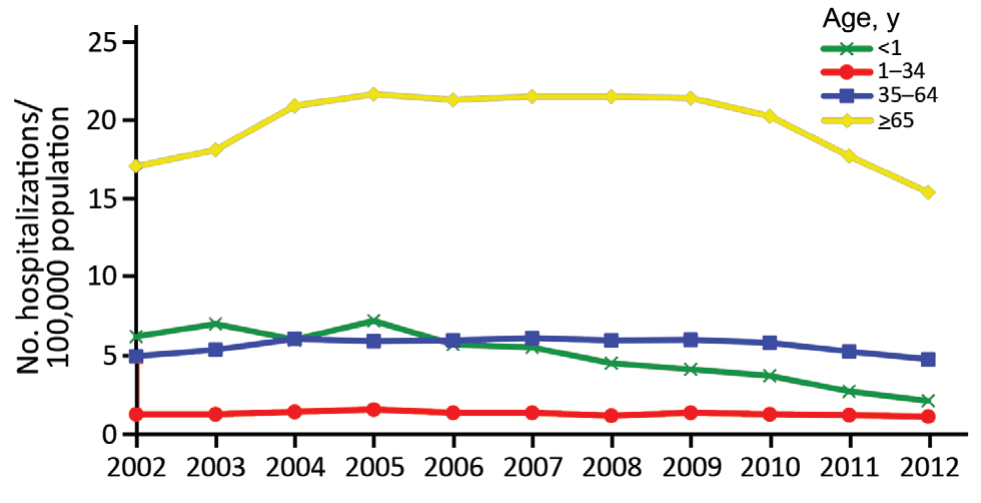
Annual rate of invasive candidiasis–associated hospitalizations by age, United States, 2002–2012. Neonates (<1 mo of age) were excluded from <1 population. Data were provided by State Inpatient Databases through the Healthcare Cost and Utilization Project maintained by the US Agency for Healthcare Research and Quality. Diagnoses were classified by using Agency for Healthcare Research and Quality clinical classification software (*17*) and multiple codes and ranges from the International Classification of Diseases, 9th Revision, Clinical Modification.

During 2002–2012, the annual age-adjusted hospitalization rate ranged from 4.3 to 5.8 hospitalizations/100,000 persons. To better describe the annual rates, we fitted a Poisson model for the period beginning in 2005 when rates appeared to be stable or decreasing. During 2005–2012, hospitalization rates decreased and showed an average APC of 4.5% for women and 3.9% for men. With the exception of persons 18–34 years of age, invasive candidiasis decreased in all other age groups during 2005–2012. The most marked decrease occurred for patients >1 month to <1 year of age; this group had an average annual decrease of 16.9% during 2005–2012 (Figure 2).

**Figure 3:**
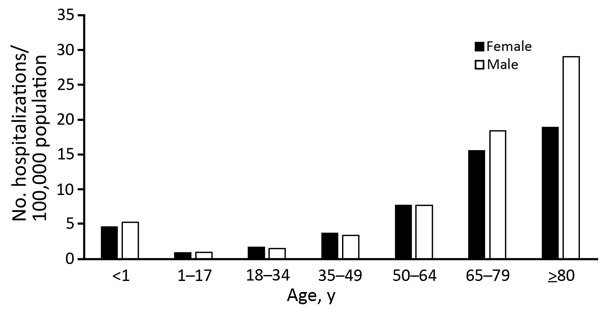
Average annual rate of invasive candidiasis–associated hospitalizations by age and sex, United States, 2002–2012. Neonates (<1 mo of age) were excluded from <1 population. Data were provided by State Inpatient Databases through the Healthcare Cost and Utilization Project maintained by the US Agency for Healthcare Research and Quality. Diagnoses were classified by using Agency for Healthcare Research and Quality clinical classification software (*17*) and multiple codes and ranges from the International Classification of Diseases, 9th Revision, Clinical Modification.

Overall, 67,432 (49%) of hospital discharges were for men, and 99,738 (72%) were for persons >50 years of age. The highest average annual invasive candidiasis–associated hospitalization rate was for persons >65 years of age (20/100,000 population), and within this group, men were at highest risk (Figure 3). For persons >34 years of age, rates appeared to double within successive age groups up to those 80 years of age. The rate for persons 50–64 years of age was 2.2-fold greater than that for persons 35–49 years of age, and overall rates for those 65–79 years of age were 2.2-fold greater than that for persons 50–64 years of age.

**Figure 4:**
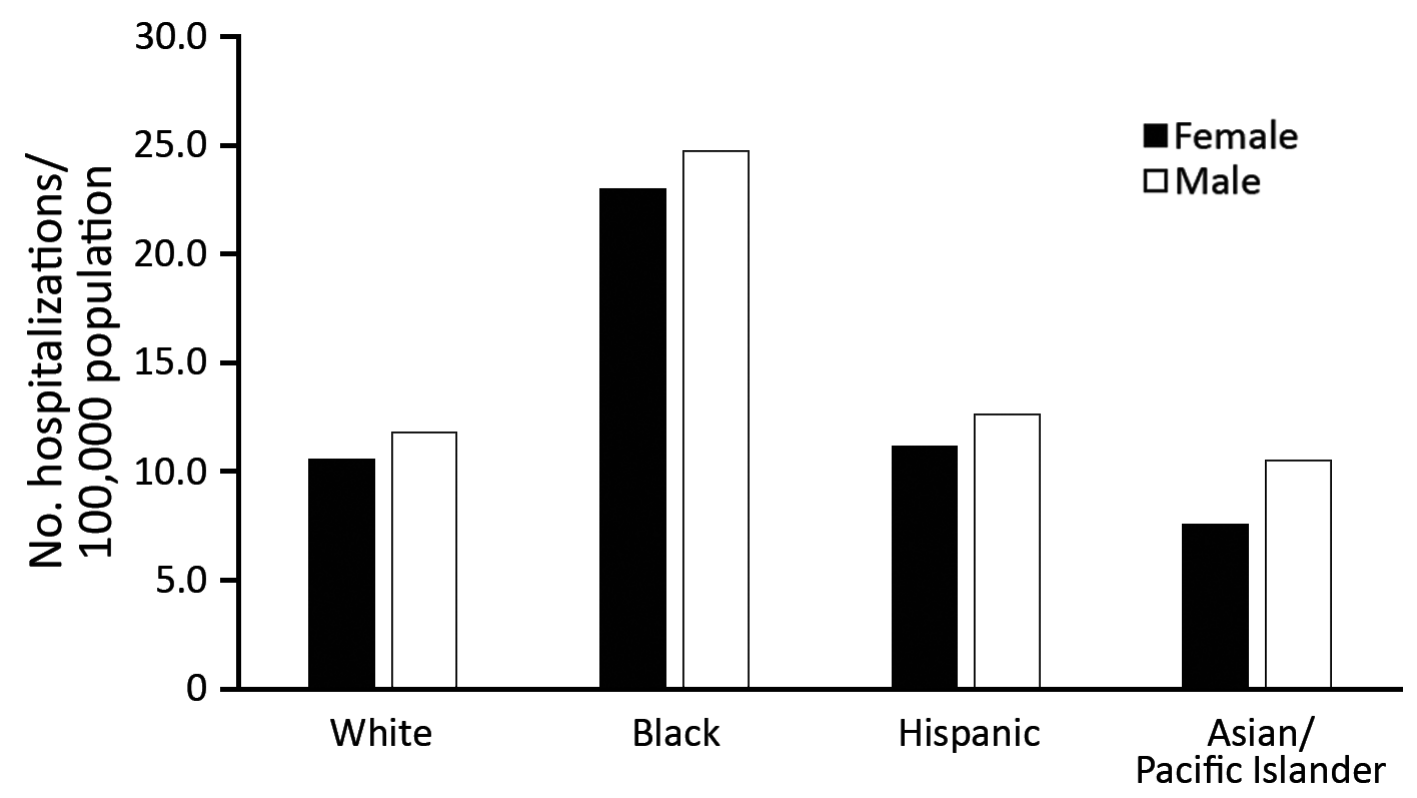
Average annual rate of invasive candidiasis–associated hospitalizations among older age groups (>50 years) by sex and race, United States, 2002–2012. Neonates (<1 mo of age) were excluded from <1 population. Data were provided by State Inpatient Databases through the Healthcare Cost and Utilization Project maintained by the US Agency for Healthcare Research and Quality. Diagnoses were classified by using Agency for Healthcare Research and Quality clinical classification software (*17*) and multiple codes and ranges from the International Classification of Diseases, 9th Revision, Clinical Modification.

To clarify racial disparities for rates, we analyzed hospitalization rates by racial/ethnic groups in age groups where incidence was highest. For persons >50 years of age, the rate for black men was 25/100,000 population, which was 2.2 times higher than that for white men. For black women, the rate was similar (23/100,000 population), which was 2.1 times higher than that for white women. Rates for Asian and Hispanic racial/ethnic groups were similar to those for whites (Figure 4). We did not find any differences in patterns of concurrent conditions by racial/ethnic group.

The most frequent underlying conditions, as a primary or secondary diagnosis, were gastrointestinal disorders or conditions (46%), hypertension (39%), diabetes mellitus (26%), and kidney disease (25%) (Table). Overall, 72% (99,360) of invasive candidiasis discharges had an ICD-9-CM code for septicemia. A total of 45% (62,092) were associated with complications of a device, implant, or graft, and 28% (38,940) were associated with complications of surgical procedures or medical care.

**Table:** Primary or secondary diagnosis for invasive candidiasis hospitalizations, United States, 2002–2012*

Diagnosis	No. (%) hospital discharges, n = 138,433
Indicator of invasive candidiasis	
Septicemia or sepsis	99,360 (72)
Complication of device, implant, or graft	62,092 (45)
Complication of surgical procedures or medical care	38,940 (28)
Underlying condition	
Gastrointestinal disorders or conditions	63,470 (46)
Hypertension	54,094 (39)
Diabetes mellitus	35,689 (26)
Kidney disease	34,626 (25)
Cancer	33,359 (24)
Congestive heart failure	30,348 (22)
Nervous system disorders	26,220 (19)
Coronary atherosclerosis and other heart disease	20,085 (15)
Chronic obstructive pulmonary disease and bronchitis	23,850 (17)
Liver diseases	19,888 (14)
Esophageal disorders	14,625 (11)

*Neonates (<1 mo of age) were excluded. Data were provided by State Inpatient Databases through the Healthcare Cost and Utilization Project maintained by the US Agency for Healthcare Research and Quality. Diagnoses were classified by using Agency for Healthcare Research and Quality clinical classification software codes (17*) *and multiple International Classification of Diseases, 9th Revision, Clinical Modification codes and ranges.

The overall median length of hospital stay was 21 days. However, the median length of stay decreased from 22 days in 2002 to 17 days in 2012, and APC decreased 1.9%. The overall in-hospital mortality rate for invasive candidiasis was 22%, although a major decrease for the in-hospital mortality rate was observed during this period (average decrease of 3.7%/year). The in-hospital mortality rate was 2-fold higher for patients >50 years of age than for those <50 years of age (25.8 vs. 13.4 deaths/100 hospitalizations for invasive candidiasis). The in-hospital mortality rate was 22% for blacks and whites.

The median cost for inpatient care in 15 states was $46,684 (range $48–$1,802,688). The median cost varied little by sex (men $48,796, range $56–$1,579,163; women $45,032, range $48–$1,802,688), but varied greatly by survival status (survived $41,096, range $48–$1,480,386; deceased $72,182, range $48−$1,802,688). The highest median costs were estimated for nonneonatal infants ($58,850) and persons 50–64 years of age ($51,447).

## Discussion

We found that state-specific rates for invasive candidiasis varied little across the United States and that hospitalizations for this disease have continued to decrease. Our overall age-adjusted hospitalization rate of 5.3/100,000 population was somewhat lower than those found previously through active population-based laboratory surveillance of candidemia during an overlapping period (2008–2011), which estimated an average annual crude incidence per 100,000 person-years of 13.3 in Atlanta and 26.2 in Baltimore (*13*). We found rates of 5.9 in Georgia and 7.1 in Maryland. The lower rates in our study are expected given that active surveillance limited to an urban area would probably detect more cases. 

Our study might have underestimated true rates for invasive candidiasis, given the limitations of administrative data, including undercoding of candidemia because of low sensitivity of blood cultures, poor provider documentation of invasive candidiasis, or discharge before receipt of laboratory results. The sensitivity of blood culture is estimated to be 50% (*21*), and culture is more likely to miss deep-seated candidiasis in the absence of candidemia. Although specific to a pediatric population, a cross-sectional analysis found that ICD-9-CM codes for candidemia had a sensitivity of 60% and a specificity >99% specific (*22*). Invasive infections might persist in organs after infections are cleared from the bloodstream, and ≈8% of candidemia cases show reoccurrence (*13*). Cultures might take 5–8 days for results to be obtained, such that patients might be discharged or die before receiving results. Thus, patients would not be coded as having candidemia, which would lead to underestimation of illness and death (*23*).

Adults >65 years of age having the highest risk for invasive candidiasis–associated hospitalization and the progressively increasing rate by age of hospitalizations among adults, with a peak among persons >80 years of age, are also consistent with a previous report of population-based surveillance for candidemia (*15*). Similarly, the 2-fold higher incidence among black persons has been reported in population-based studies of candidemia in Atlanta and Baltimore. The reasons for this racial disparity are not fully understood. A recent study conducted in 4 US cities found that adjusting for poverty attenuated the association of black race with candidemia; however, a persistent 2-fold racial disparity remained even after this adjustment (*24*).

The decrease in hospitalizations for invasive candidiasis and deaths from this disease across nearly all age groups is consistent with results from other studies that used similar time frames. Cleveland et al. also reported a major decrease in these parameters in Atlanta and Baltimore during 2008–2013 (*15*). A major cause of bloodstream infections is central line–associated bloodstream infections. Cleveland et al. found that 85% of candidemia patients had used a central venous catheter <2 days before the bloodstream infection culture date (*15*). Estimates from the Centers for Disease Control and Prevention (Atlanta, GA, USA) identified a marked decrease in central line–associated bloodstream infections during 2001 and 2008–2009. These decreases were attributed to increased state and regional prevention efforts supported by several federal agencies after establishment of a national goal in 2009 to reduce central line–associated bloodstream infections by 50% by 2013 (*25*).

Few studies have reported on length of stay and associated trends among invasive candidiasis–related hospitalizations. A recent US study reported a mean length of hospital stay of 22 days for persons with candidemia by using the Surveillance and Control of Pathogens of Epidemiologic Importance database (*3*). We report a median length of stay of 21 days and a major decreasing trend during 2002–2012. A study in 2005 used the AHRQ 2000 Nationwide Inpatient Survey for 28 states to analyze attributable outcomes among adult patients (>18 years of age) with hospital-associated candidemia (*26*). This study reported a mean estimated length of stay of 18.6 days and associated charges of $66,154 in 2000 US dollars. Our reported median hospitalization costs of $46,684 for invasive candidiasis–associated hospitalizations are lower. However, charges probably overestimate actual costs. In addition, median costs limit data extremes from skewing results and provide greater accuracy. Finally, our costs reflect 11 years of hospital discharges, include nonneonatal hospitalizations of patients <18 years of age, and reflect multiple invasive candidiasis codes.

*Candida* species remain the leading fungal cause of healthcare associated infections and the seventh most common overall pathogen, representing 6% of all healthcare-associated infections; in 2011 an estimated 648,000 patients had >1 healthcare-associated infection, representing 4% of all inpatients in the United States (*4*). Although national invasive candidiasis–associated hospitalization rates have been decreasing for men and women since 2005, the incidence of invasive candidiasis–associated hospitalizations remains high and is associated with substantial mortality rates and health costs. Continued research is needed to identify interventions associated with these decreasing trends to further accelerate this observed decrease, including improved prevention and treatment, such as optimum antifungal treatments and timing of medical procedures.
